# Intrusion detection system for V2X communication in VANET networks using machine learning-based cryptographic protocols

**DOI:** 10.1038/s41598-024-82313-x

**Published:** 2024-12-30

**Authors:** Thiruppathy Kesavan Venkatasamy, Md. Jakir Hossen, Gopi Ramasamy, Nor Hidayati Binti Abdul Aziz

**Affiliations:** 1 Faculty of Information Technology, Dhanalakshmi Srinivasan Engineering College, Perambalur, Tamil Nadu India; 2 Faculty of Engineering and Technology, Multimedia University, Melaka, Malaysia; 3 Faculty of Computer Science & Engineering, Dhanalakshmi Srinivasan Engineering College, Perambalur, Tamil Nadu India; 4 Postdoctoral Fellow, Engineering and Technology, Multimedia University, Melaka, Malaysia

**Keywords:** V2X communication, Vehicular ad-hoc networks, Machine learning, Intrusion detection system, Cryptography, Information technology, Computational science

## Abstract

Vehicle-to-everything (V2X) communication has many benefits. It improves fuel efficiency, road safety, and traffic management. But it raises privacy and security concerns. These include the risk of cyberattacks and the loss of drivers’ personal data. Eavesdropping, data manipulation, and unauthorized vehicle monitoring are major problems that need immediate attention. This paper proposes a new approach to intrusion detection in V2X communications. It uses machine learning-based cryptographic protocols for intrusion detection (ML-CPIDSs). The goal is to improve privacy and security in vehicular ad hoc networks (VANETs). The ML-CPIDS combines advanced cryptographic protocols with machine learning. It provides strong authentication, encryption, and real-time threat detection. Robust authentication and encryption techniques in modern cryptographic systems protect sensitive information. Using machine learning algorithms, it is feasible to identify and address security risks in real-time. The proposed technology solves key privacy and security issues. It has applications in many areas, including autonomous vehicle networks, urban traffic management, and vehicle communication systems. Extensive simulations show the ML-CPIDS works in different VANET environments. Privacy, security, and the ability to identify threats in real time are some of the areas that are evaluated in these simulations. The proposed ML-CPIDS approach outperforms current methods on several metrics. It has better privacy and authentication, lower latency, and stronger threat detection. It also improves the integrity and efficiency of V2X communications in VANET networks.

## Introduction

VANETs and V2X connections have transformed intelligent transportation systems forever^1^. Traffic safety, congestion, and road connections depend on cars’ ability to interact with each other and their surroundings^2^. V2X communication has many benefits. But, serious privacy and security issues remain^3^. This may be subject to the fragility of drivers’ personal information and the opportunity to expose it to attackers^4^. It is critical to prevent data tampering, eavesdropping, and illegal vehicle surveillance. This will ensure the steady, nonstop functioning of VANETs^5^. The ML-CPIDS is a new method proposed in this paper to solve the abovementioned issues^6^. By integrating current cryptographic protocols with machine-based strategies, the ML-CPIDS improves the confidentiality and integrity of V2X communication^7^. This integration improves encryption, authentication, and real-time threat detection^8–11^.

Autonomous car networks, city visitor management, and car communication systems may also all use their all-encompassing answers^12^. Cyberattacks and information breaches are a long way and much less likely to appear if the advised solution ensures the privacy and security of the data sent to the community^13^. A comprehensive simulation evaluation is frequently executed to illustrate the efficacy of the ML-CPIDS. With an emphasis on privacy, protection, and real-time hazard detection metrics, this simulation evaluates the device’s capabilities under numerous VANET conditions^14^. The findings indicate that ML-CPIDS has promise as a dependable answer to the complex problems associated with VANET networks, particularly in enhancing the privacy and security of V2X interactions^15^. Against different attempts to protect VANETs, the ML-CPIDS device stands head and shoulders above the competition because it integrates cryptographic protocols and device information.

The weaknesses in VANET intrusion detection techniques that rely on machine learning and cryptographic protocols must be addressed. The security measures have been enhanced; however, problems with communication protocol incompatibility, dynamic vehicle settings, and insider threats still exist. New evidence shows that static models cannot handle dynamic threats. So, conventional thinking is not enough. The ML-CPIDS architecture, which is the subject of the proposed research, employs state-of-the-art machine learning and adaptive cryptographic protocols to overcome these problems. ML-CPIDS provides stronger security than previous systems by reducing insider risk via behavioral analysis and real-time anomaly detection. Standard communication protocols make it interoperable across various automobile manufacturers. VANETs are becoming more complex and diverse, requiring a security solution that can adapt to new threats and increase V2X communication dependability and safety.

Data scarcity and privacy issues trouble ML-CPIDS model training for VANETs. Evolving attack trends further complicate this process. Outdated information fails to counter dynamic threats effectively. These challenges demand innovative approaches to secure vehicular networks. This is solved by continuous model retraining employing online and transfer learning for real-time threat adaptability. The use of federated learning ensures the security of vehicle data while making use of real-world data. Edge computing and lightweight models collaborate to efficiently use available resources, whereas anomaly detection and data augmentation address updated datasets. These strategies enhance the model’s performance and detection accuracy in dynamic threat environments.

The architecture’s adaptability makes safe integration with various vehicles and communication networks possible. Adaptive machine learning models, which can utilize several types of data, are used to detect threats in different network environments. Following these standards and using flexible design principles allows ML-CPIDS to link infrastructure and vehicles easily, regardless of the protocol or manufacturer, while maintaining security.

The contributions of this research are as follows:

- The ML-CPIDS system uses machine learning with cryptographic protocols. It improves real-time threat detection and privacy protection. This addresses gaps in existing automotive network security systems.
- Data aggregation and anonymity protect sensitive info, like vehicle locations and identities. The system’s real-time anomaly detection algorithms find security issues.
- The ML-CPIDS can adapt to changing VANETs. It guarantees scalability and high performance as vehicles move in the network.

The rest of the paper is arranged as follows: Sect. 2 explains the related works, Sect. 3 explains the proposed method of the article, Sect. 4 explains the results and discussion, and finally, Sect. 5 explains the paper’s conclusions and future extensions.

### Related works

To improve the privacy and security of CT-driven V2X applications, an integrated blockchain and deep learning architecture is used^16^. This architecture ensures secure communication through smart contract-based Proof-of-Work and Zero Knowledge Proof. Compared with other methods presently in use, it performs better in terms of communication and security. With fast wireless communications and autonomous vehicle development, intelligent and autonomous automobiles will be implemented quickly. The security and management of 5G V2X might be strengthened via blockchain and multiaccess edge computing (MEC). Blockchain integration in 5G-based MEC vehicular networks is analyzed for security, privacy, and content storage. It discusses edge computing and 5G V2X concerns, challenges, and future research paths in these integrated and developing technologies.

V2X on 5G wireless networks is the only technology that can satisfy autonomous vehicle criteria for low latency and reliable communications in highly mobile surroundings^17^. This technology is gaining importance because it meets these needs. This system proposes a 5G-based secure vehicle-to-vehicle communication system that complies with these criteria. Pro verification has tested its infrastructure against Vehicle-to-Vehicle (V2V) attacks and has revealed that it is secure. This demonstrates that the method fulfills autonomous driving end‒to-end latency criteria and is more efficient than previous techniques when performance assessment findings are used.

Reference^18^ presents a centralized adaptive pseudonym change scheme that enables vehicles to request pseudonym changes from the certificate holder. This approach responds to traffic density and privacy settings, helping to prevent Sybil attacks and minimizing redundant pseudonym usage. Finding an answer to the trade-off problem is its primary objective. It uses an algorithm rooted in the knapsack problem for target tracking and an entropy-based method for identifying each vehicle’s privacy level. The analyses show that the proposed approach can accurately indicate the level of secrecy; thus, it could be the best reaction to changing the pseudonyms.

A novel privacy-preserving technique^19^ using edge-based computing is presented in this paper. This protocol aims to make transportation networks safer. It presents iCLASC, an improved certificate-less aggregation sign-cryption method that uses the information transfer protocol for road monitoring data. The aim is edge processing data security; privacy, anonymity, joint authentication, secrecy, and integrity are key security protocol components. The authors proposed an improved intrusion detection system based on support vector machines and an autoencoder network^20^. The objectives are to identify the five primary forms of attacks Deniel of Service (DoS), distributed DoS (DDoS), Wormhole, black hole, and gray hole attacks) that VANET might encounter by fusing the power of autoencoder feature extraction with SVM capacity to exploit massive volumes of data.

The CyberTwin (CT) architecture is a communication and digital asset owner that can make the V2X network safe and versatile^21^. To improve privacy and security in CT-driven V2X applications, this paper introduces BDTwin, an integrated framework based on blockchain technology and deep learning. A blockchain system that employs a smart contract-based enhance-proof-of-work and zero-knowledge proof verification method achieves secure communication between automobiles, roadside units, CT-edge servers, and cloud servers. Experimental findings and security analysis utilizing two sources demonstrate that the proposed BDTwin architecture outperforms the baseline and state-of-the-art methods.

Marwah et al. introduced an integrated architecture based on the Internet of Things to improve security and privacy in V2X applications driven by CT, inspired by the previously discussed topic^22^. An IoT system is intended to provide safe communication between automobiles, roadside apparatuses, CT-edge servers, and cloud servers through an enhanced proof-of-work and zero-knowledge proof verification procedure based on smart contracts.

Zhang et al. proposed a novel machine learning method^23^ to enhance VANET performance. With cutting-edge machine learning technology that yields vehicle information, the suggested solution detects DDoS attempts. The suggested approach integrates alternate data transmission channels and the 5G cellular network. Over three months, the data collection experiment was conducted at several locations throughout Berlin and involved the use of 5G technologies with Big Data^24^ assistance from position-based routing protocols. The methods were used to identify nonline-of-sight scenarios instantly, ensuring secure data transfer without compromising network performance. This study is new because it addresses a variety of traffic scenarios (the degree of traffic congestion can impact the quality of big data transmission) and provides a method for enhancing big data transfer via 5G technology.

By combining a transformer, federated learning, and the Paillier cryptosystem, this study proposes an effective federated deep learning-based technique for detecting false data injection attacks (FDIAs)^25^. As an edge node detector, the Transformer uses its multiheaded self-attention mechanism to examine the relationships among certain electrical variables. This approach makes use of a federated learning framework to train a detection model together using data from all nodes, all while protecting data privacy by storing the data regionally during training.

An innovative security risk to the reliable functioning of power systems is the fake data injection attack (DDIA)^26^. This research takes a thorough approach to address this problem. The paper begins by examining the fundamentals of these new DDIAs and how these affect power systems. To account for attacker-perspective alternating current (AC) state estimates and insufficient topological information, a detailed mathematical model of the DDIA is constructed here. The objective of this approach is to reduce the risks associated with attack data generation techniques that use direct current (DC) flows and are easy to detect.

With the latest advancements in Cellular Vehicle-to-Everything (C-V2X) technology, vehicles can seamlessly communicate with one another using both direct and network communication methods. The paper^27^ introduces an innovative vehicle-to-vehicle communication system (BVCS) that is enhanced by blockchain technology, significantly improving the security of both automobiles and the data shared between them. By implementing smart contracts, the authors demonstrate how automated user and vehicle authentication can be achieved, ensuring that only authorized individuals gain access. The methods outlined in the paper not only authenticate users and detect unauthorized access but also establish robust and secure communication channels between vehicles, paving the way for safer and more efficient transportation.

A blockchain-based sustainable safety management system for linked automobiles was suggested in^28^. An AI-enabled vehicle smart device (AVSD) for vehicular communications has been introduced as smart transportation equipment. By lowering the computational expenses of vehicle communications, AVSD could decrease energy usage. Vehicles could be automatically identified, and secure communication could be established between them and emergency service stations (ESSs) such as hospitals, police stations, and fire stations using smart contracts. The findings of the experiments demonstrate that the suggested framework establishes a communication environment for long-term protection and security via the use of the developed smart transportation devices.

The advancement of ITSs has driven road condition monitoring systems primarily via V2X communication based on 5G. Table 1 summarizes the findings from the literature. However, issues with processing delays and network latency in the existing architecture limit the use of available bandwidth. To strengthen privacy and security in V2X communication for VANET networks, this research suggests a new method, ML-CPIDS.

**Table 1:** Summary of various methods discussed in the literature.

S. No	Methods	Advantages	Limitations
1	Multiaccess Edge Computing (MEC)^16^	- Reduces scalability and flexibility issues by bringing cloud services closer to vehicle nodes - Improves latency for real-time applications - Enhances security and privacy in vehicular networks	- Dependency on edge node reliability - Initial deployment costs - Potential for increased network complexity
2	Blockchain in 5G-based MEC systems^17^	- Enhances security, privacy, and networking challenges in-vehicle networks - Provides decentralized trust and tamper-proof data records -Facilitates secure transactions and data sharing among vehicles	- High computational overhead - Scalability challenges -Integration complexity with existing infrastructure
3	Secure 5G-based Vehicle-to-Vehicle (5G-V2V)^18^	- Meets low latency and reliable communication requirements for autonomous vehicles - Uses Network Slicing (NS) for efficient resource allocation - Enhances security against V2V attacks	- Requires robust network infrastructure -Potential interoperability issues - Cost of implementing NS and security measures
4	Centralized Adaptive Pseudonym Change Scheme^19^	- Improves privacy by dynamically changing vehicle identifiers - Resilient against Sybil attacks - Responds to changes in traffic density and user privacy settings	- Centralized nature may pose a single point of failure - Overhead in managing pseudonym changes - Potential latency in the pseudonym update process
5	BDTwin (Blockchain and Deep Learning)^21^	- Integrates Blockchain and Deep Learning for enhanced security and privacy in V2X applications -Facilitates secure communication and data management - Outperforms traditional methods in security analysis	- Complexity in integrating Blockchain and Deep Learning technologies - High initial setup and maintenance costs - Requires specialized expertise in both domains
6	Intrusion Detection System (IDS) using SVM and autoencoder network^22^	- Effectively identifies various attacks (DoS, DDoS, Wormhole, etc.) in VANETs - Utilizes advanced machine learning for anomaly detection - Enhances network security and reliability	- Requires significant computational resources - Dependence on accurate training data for effective intrusion detection - Complexity in fine-tuning IDS parameters for specific VANET environments
7	IoT-driven security architectures^23^	- Improves overall security and versatility in V2X applications - Distributes digital assets securely among entities -Supports safe communication between various network components	- Integration challenges with diverse IoT devices - Potential scalability issues with growing IoT networks - Complexity in managing distributed security policies across IoT nodes
8	Novel Machine Learning Method^24^	- Enhances VANET performance by detecting DDoS attempts using machine learning - Minimizes communication overhead and latency - Improves security against network intrusions	- Dependence on accurate and up-to-date machine learning models - Initial setup and training costs - Potential false positives or negatives in intrusion detection - Complexity in integrating with existing VANET infrastructure
9	Integration of big data transmission channels and 5G^29^	- Utilizes 5G cellular networks for enhanced data transmission speed and reliability - Integrates with position-based routing protocols for efficient data delivery - Addresses non‒line-of-sight scenarios for improved network coverage	- Requires substantial investment in 5G infrastructure - Compatibility challenges with existing VANET protocols - Potential for increased electromagnetic interference in dense urban environments - Regulatory and licensing issues with deploying 5G networks

### Proposed intrusion detection system for V2X communications in VANETs

The V2X interaction, which allows for real-time communication between vehicles and their environments, has extended VANETs. To improve the user protection, effectiveness, and connection, this era is essential for advancing smart transport systems^3^. Despite those benefits, V2X communication poses critical risks to drivers’ privacy and protection, along with the possibility of hacks and the disclosure of sensitive data. Addressing issues such as data manipulation, eavesdropping, and illegal vehicle monitoring is crucial. ML-CPIDSs have been proposed to strengthen privacy and security in V2X communication for VANET networks. The goal is to reinforce V2X communications inside VANETs.

### Framework for reliable and secure communication in VANETs

**Fig. 1:**
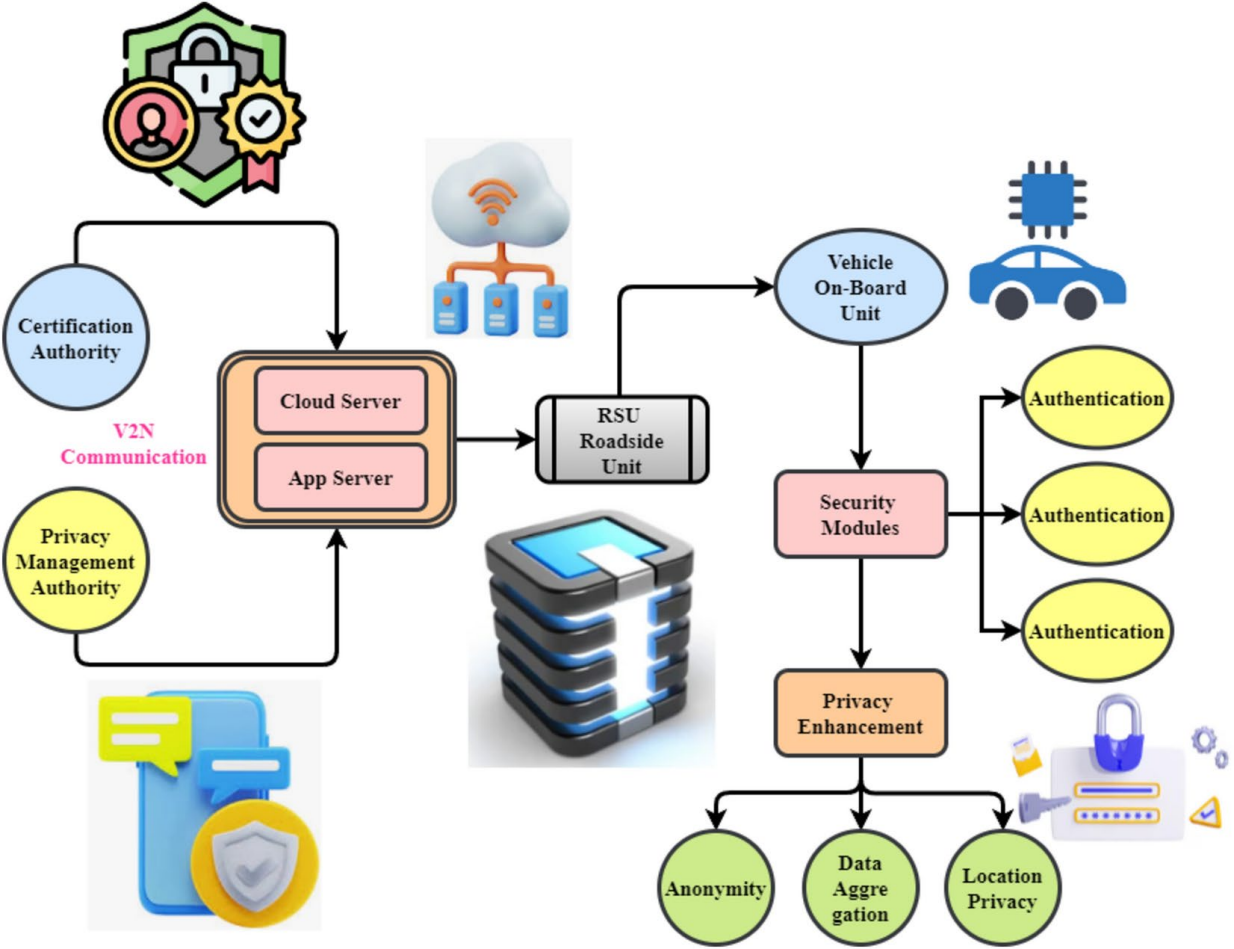
Framework for the proposed IDS for VANETs.

Figure 1 shows the structure that was developed for an IDS to improve the reliability and security of VANETs through V2X communication. To protect data and ensure effective processing, architecture uses several layers of connectivity and security protocols. It depends on the authorities, internet-based services, monitoring devices, and internal vehicles^4^. The Certification Authority and the Privacy Management Authority are the two branches of the Trusted Authority at the best level.

The ML-CPIDS concept uses multiple elements to securely transport data between cars. The cloud server is a central hub which processes, stores, and analyzes data from cars and RSUs. The app server supports various vehicle communication apps, including navigation, traffic monitoring, and intrusion detection. Trust and privacy are built via the Certification Authority. Privacy improvement protects user data via data aggregation, anonymization, and location privacy. Real-time data analysis, proactive risk detection, car network communication management, and personal data protection are possible with its complete security architecture. The IDS’s ability to detect risks is assessed through a structured modeling approach. Algorithms are developed to distinguish between normal and malicious network activities. By analyzing traffic patterns and spotting irregularities, the IDS quickly detects suspicious behavior.

**Fig. 2:**
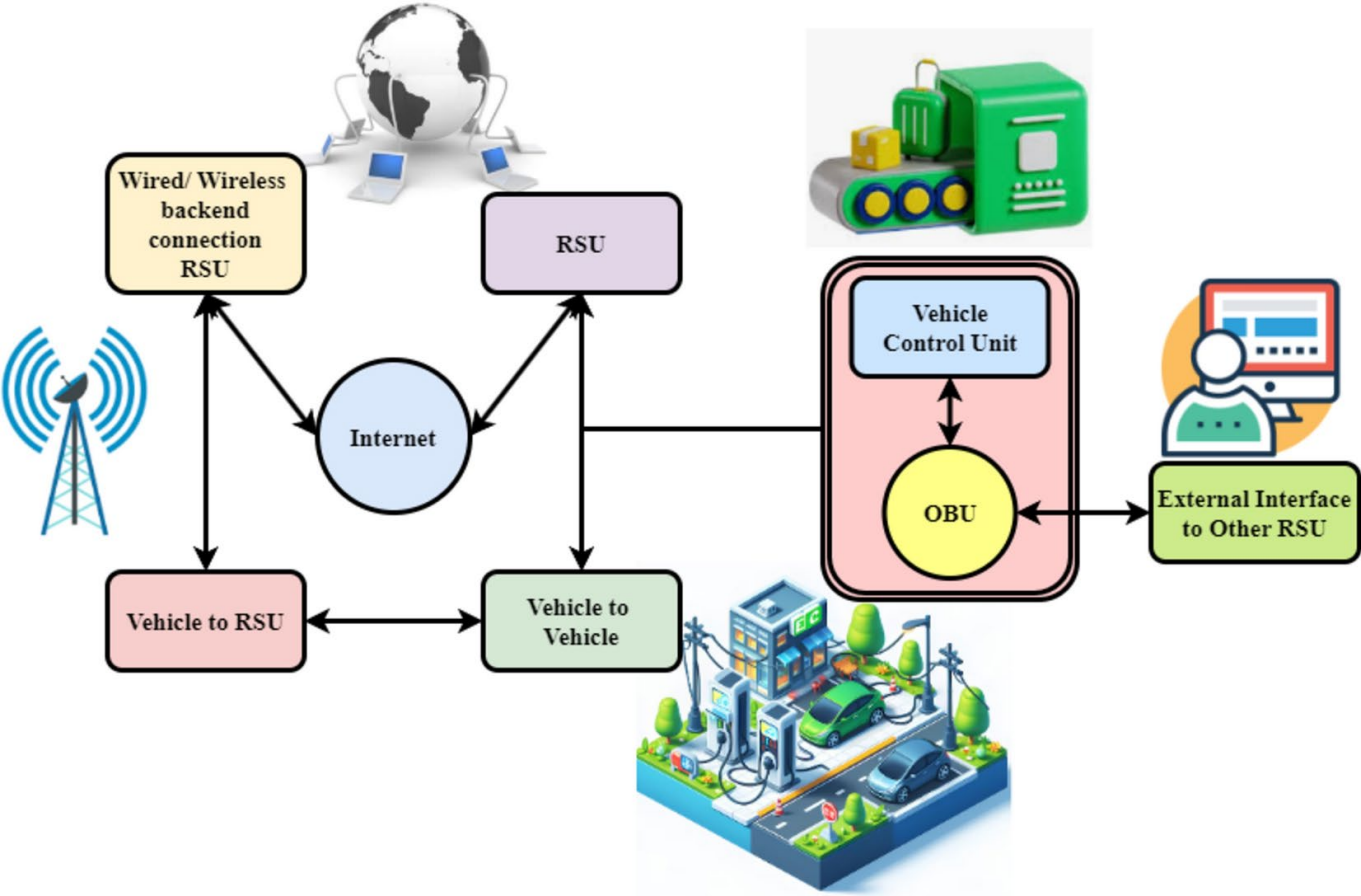
Secure V2X communication platform.

V2X ecosystem exchange methods and ability applications have been thoroughly examined in^12^. The safety of the V2X connection is shown in Fig. 2, which represents how VANET Vehicle Control Units (VCUs) communicate with cars, the roadside infrastructure, and other services. Real-time V2V data from the VCU and on-board units enable collision avoidance and enhance security. Vehicles and RSUs communicate simultaneously, sharing sensitive information. RSUs connect to the network via wired or wireless internet, facilitating seamless data exchange. RSUs connect automobiles to cloud services for remote monitoring, real-time traffic control, and system integration. The internet connects everyone, improving vehicle network efficiency and security. The VCU extends the network and speeds up vehicle-to-RSU zone transfers by communicating with other RSUs. Integrated system design enhances vehicle communication and supports autonomous and intelligent mobility.

VANETs use advanced networking and intelligence methods. Traditional VANETs are Intelligent Transportation Systems (ITS) subsystems since they focus on V2V communication and connection protocols provided by IEEE 802.11p and cellular technologies. VANETs usually have limited internet connections; thus, they are used mostly for traffic efficiency and user safety^21^. Intelligent cars with onboard sensors, processing, and storage platforms may connect with any entity via V2X protocols, the future Internet of Things paradigm.

**Fig. 3:**
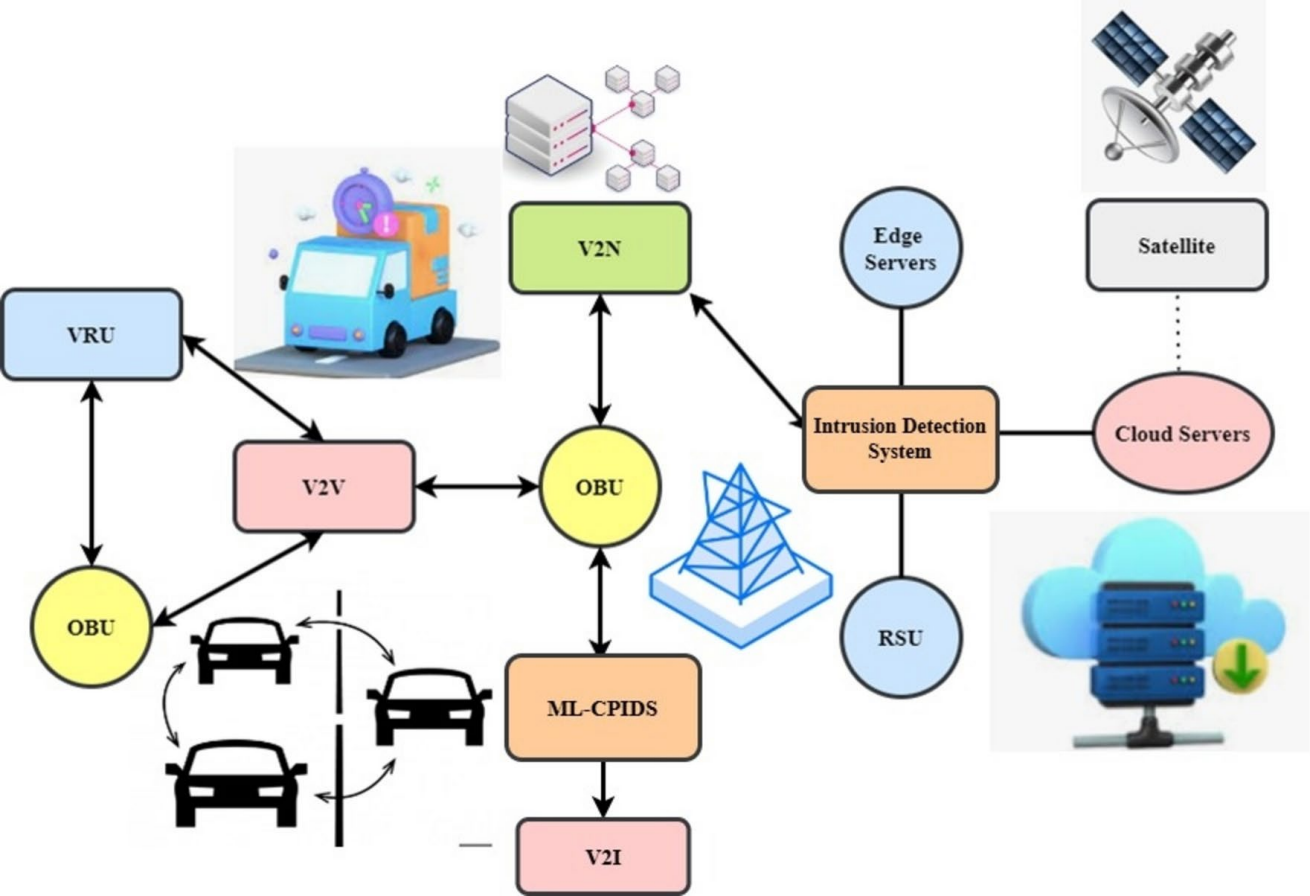
Connectivity of nodes in the V2X network.

Figure 3 illustrates a V2X communication architecture involving V2V, Vehicle-to-Infrastructure (V2I), and Vehicle-to-Network (V2N) interactions. It connects On-Board Units (OBU) within vehicles to various external systems, such as edge servers, cloud servers, and Intrusion Detection Systems (IDS), enhancing safety and efficiency in smart transportation networks.

- V2V and V2I Communication: Vehicles equipped with OBUs communicate with each other (V2V) and with infrastructure elements like roadside units (RSU) in the environment (V2I). This communication helps coordinate vehicle movements and improve traffic flow, preventing accidents.
- V2N Communication: The system also enables V2N communication through cellular networks and satellites, connecting vehicles to remote cloud and edge servers. This supports real-time data processing, such as for navigation, updates, or hazard warnings.
- ML-CPIDS: This system provides security by analyzing vehicle and network data to detect malicious activities or cyber threats, ensuring secure V2X communication.

The interconnected components form a holistic framework, enhancing vehicle and road safety while leveraging cloud-based processing, edge computing, and satellite support for efficient and secure communication.

**Fig. 4:**
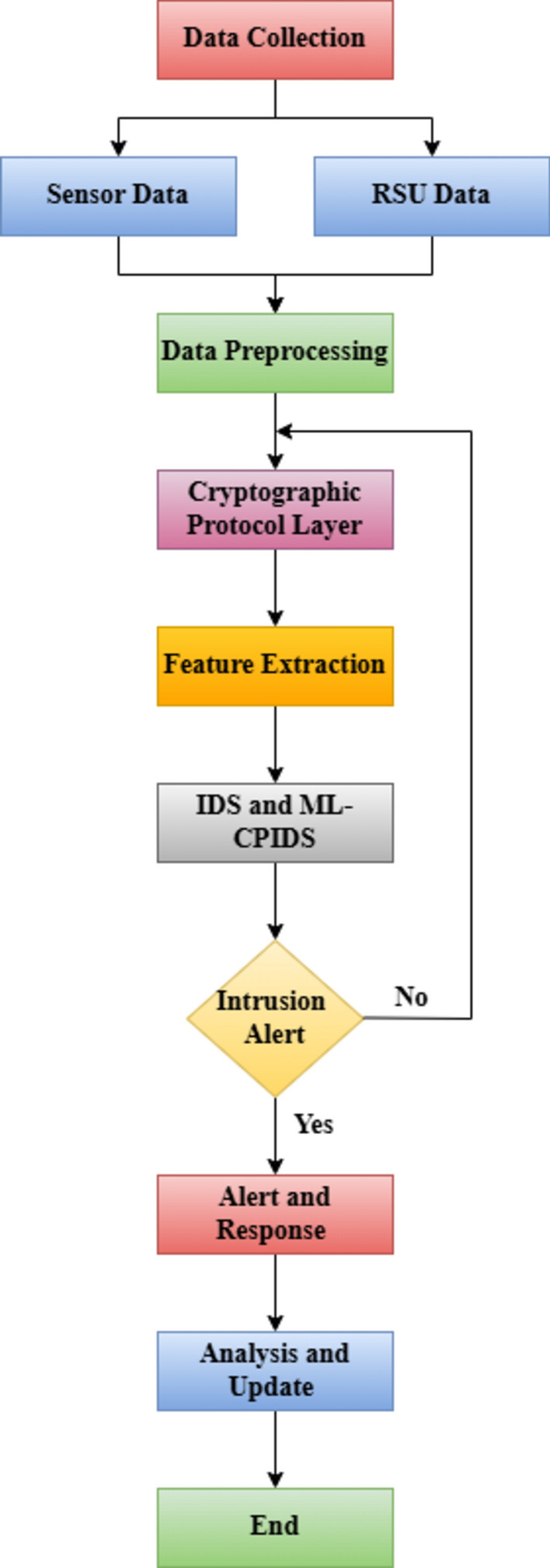
Workflow of proposed IDS and ML-CPIDS.

Figure 4 shows the flow diagram of the proposed ML-CPIDS that uses cryptographic protocols based on machine learning to track V2X traffic on VANET networks in real-time. An important first step in network monitoring is data collection from a variety of sources, such as vehicle sensors and RSUs. It goes through data preprocessing after data collection to make sure everything is consistent and to reduce noise. The data subsequently moves on to the cryptographic protocol layer, where encryption is used to protect the information from illegal access while keeping it intact and private.

The feature extraction process entails the encrypted data for useful properties, including the latency and frequency of communications, that can be used to identify typical or unusual behavior. ML-CPIDS uses machine learning models to identify abnormalities or possible intrusions when the processed data is provided to it. After an intrusion alert arises, the system goes through a sequence of alert and response methods to deal with potential dangers, such recording the event or alerting the administrators. If no unusual behavior occurs, the system returns to the beginning of the data analysis cycle.

Following a response’s execution, the system can gain information from identified threats at the analysis and update step. This enables the machine learning model to be updated, resulting in improved accuracy and the ability to respond to new threat patterns. At the end of this statement, the IDS is ready to keep an eye on and protect V2X communication. A strong defense for VANET networks is ensured by this flow, which allows the system to adapt and react continually to emerging security threats.

### Intrusion detection system for enhanced security in VANETs

The V2X communication structures are made possible via advanced vehicular Wi-Fi technology, allowing motors to share data in real time, irrespective of the vicinity or network. By facilitating communication among vehicles, infrastructure, people, and networks, this ability greatly complements the ability to handle visitors, protect, and drive satisfaction. V2X apps provide valuable benefits, but they present significant privacy risks. They can enable unauthorized tracking and data theft, especially if car networks and apps are compromised. Protecting your privacy is essential.

1 $${{q}_{j}e}_{jq} = <qj{e}_{jk}^{1}, qj{e}_{jk}^{2}> = {\forall }_{1}Q, J{E}_{wj} \pm {i}_{1}\left({\partial }_{1}{Q}_{qrc}\right)$$

Encryption (${{q}_{j}e}_{jq}$), robust authentication ($qj{e}_{jk}^{1}$), and real-time identifying threats ($qj{e}_{jk}^{2}$) are created by the interaction between the encrypted data (${\forall }_{1}Q$) and the cryptographic protocols ($J{E}_{wj}$). With respect to privacy and security in communication over V2X and ${i}_{1}\left({\partial }_{1}{Q}_{qrc}\right)$ for VANETs, Eq. (1) sums up the holistic approach of ML-CPIDS.

2 $${\forall }_{p}\left(q+p\right)= {i}_{2} (qj{e}_{jk}^{1}|\left|qj{e}_{jk}^{2}\right){Q}_{qrc}+ {Z}_{jk}\left(m+qr\right)$$

In Eq. (2), ($qj{e}_{jk}^{1}|\left|qj{e}_{jk}^{2}\right)$ is integrated with the data value (${Q}_{qrc}$)^5^. It emphasizes how these components work together to improve security by integrating cryptographic power (${Z}_{jk}$) and the ability to identify threats in real time and by guaranteeing the safe processing of each data point ($m+qr$).

3 $$\left(\sum_{j=1}^{p}\left({\forall }_{j}+{\partial }_{np}\right)\right)+Q=\left(\sum_{j=1}^{p}\left({\propto }_{j}+{j}_{2}\right)+\left(qj{e}_{jk}^{1}|\left|qj{e}_{jk}^{2}\right)\right){Q}_{qrc}\right)$$

When the element $Q$ is combined with the Eq. (3) term, which represents various security parameters (${\forall }_{j}+{\partial }_{np}$), the result is an improvement in the detection of threats ${\propto }_{j}+{j}_{2}$ and responses. The integration of (${Q}_{qrc}$) and cryptographic protocols ($\left(qj{e}_{jk}^{1}|\left|qj{e}_{jk}^{2}\right)\right)$)) guarantees complete privacy and security in V2X communication for VANETs.

4 $${\beta }_{nj}. Q= {i}_{j,2} {Q}_{yrc}+\left({\alpha }_{nj}Q-{j}_{j,2}Q-{Q}_{qrc}\right)= {\alpha }_{nj}Q$$

The proposed intrusion detection method employs a machine learning algorithm that encompasses several key stages: data collection, feature extraction, and hashing for message authentication. To improve the efficiency of detecting and reducing possible risks in the network, trust calculation, training, and intrusion detection functions are also combined in the algorithm. An overview of the algorithm is given in Algorithm 1.

This approach begins with data collection and simulation of messages from vehicles tagged as normal and malicious. After that, the feature extraction identifies the required properties such as the timestamp and signal strength and the occurrence of the binary label on the messages. A hashing function is used for the authentication of the messages. Indirect trust calculation is used to estimate the involved trust levels among observers, whereas position closeness is used to evaluate the closeness of the nodes. The training data are split into training and fitting a model for machine learning. The communication cost is computed for data transfer, which leads to intrusion detection, where the model predicts anomalies in incoming data.

**Algorithm 1:**
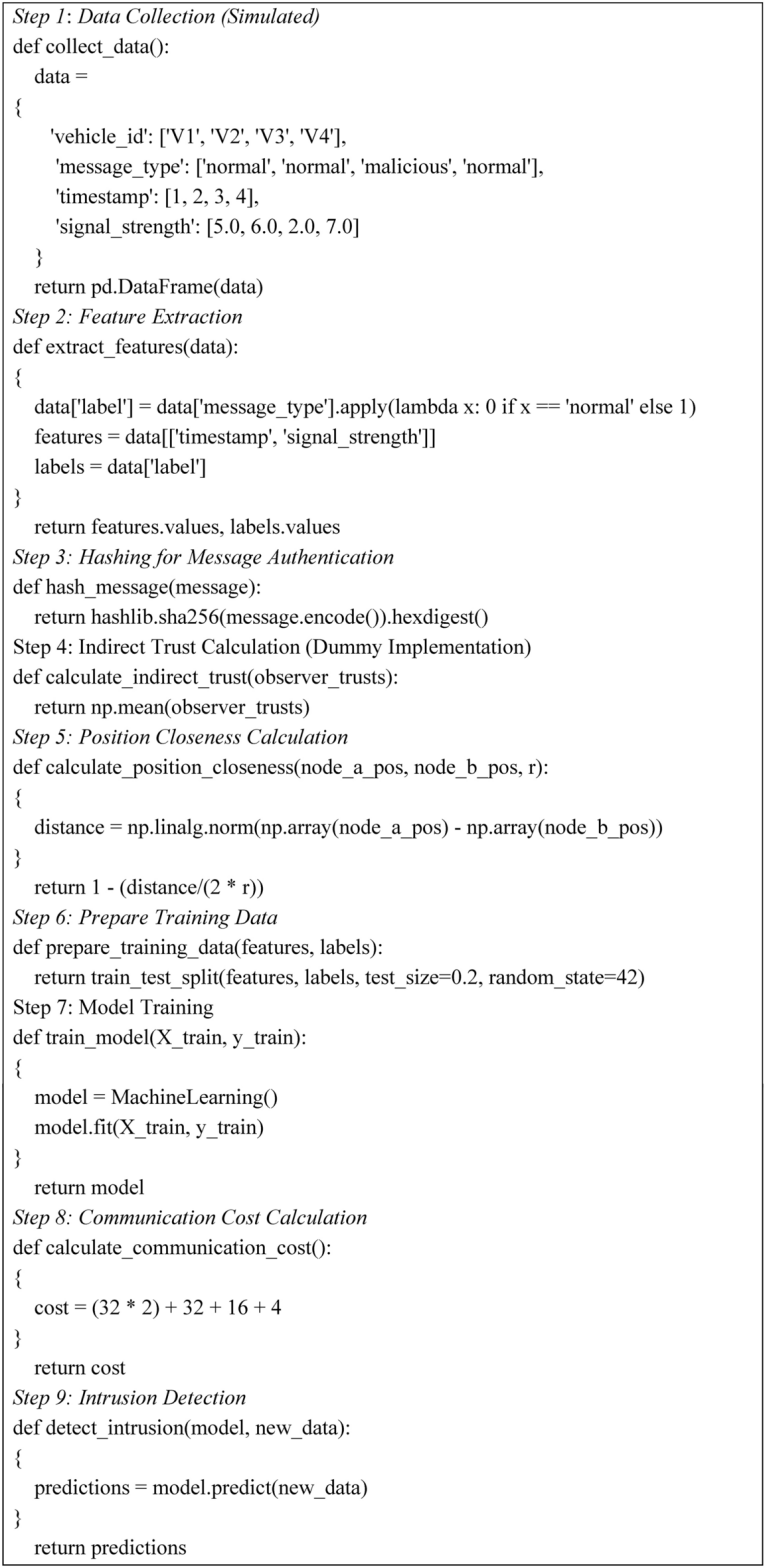
Process of proposed method using machine learning.

ML-CPIDS enhances real-time data processing in heavy traffic by using edge computing to analyze data closer to the source, reducing latency and bandwidth use. It employs lightweight, optimized machine learning models for quick and efficient analysis. By filtering data, ML-CPIDS prioritizes key information and processes data from multiple vehicles simultaneously. Adaptive learning further improves threat detection based on new data, and a distributed computing setup allows parallel processing across nodes. Together, these strategies enable ML-CPIDS to efficiently process high volumes of data while accurately detecting threats.

Data manipulation, eavesdropping, illegal use, and major cyberattacks threaten V2X privacy and security. IDS, encryption, and privacy-boosting technologies are suggested for V2X security. Identifying semantic gaps in these safety measures reveals defensive vulnerabilities and potential hazards.

### Examining security dimensions

Cryptographic protocols are essential in IDSs for VANETs to guarantee data integrity and secure communication. These protocols are designed to protect against illegal access and the alteration of private data that are sent between infrastructure and automobiles. The security analysis evaluates various parameters, including encryption strength, key management efficiency, strength, resilience, integrity, privacy, threat detection and accuracy. This comprehensive approach to security analysis is essential for maintaining trust and safety in intelligent transportation systems.

5 $$\left({\propto }_{kp}- {\infty }_{{n}_{j}}^{*}\right)Q={\propto }_{kp}Q- {\propto }_{kp}*Q \left(v-{vp}^{2}\right)+\left(m+rst\right)$$

Equation (5), $\left({\propto }_{kp}- {\infty }_{{n}_{j}}^{*}\right)Q$, represents the standard level of protection offered by cryptographic protocols, but the expression ${\propto }_{kp}Q$ takes into consideration differences and possible weaknesses in in-vehicle communications ${\propto }_{kp}*Q \left(v-{vp}^{2}\right)$. The machine learning component’s contributions to improving security are represented by the inclusion of $\left(m+rst\right)$.

6 $$FU= \frac{1}{N} \sum_{j=1}^{w}N \left({U}_{out}^{j}- {U}_{in}^{j}\right)+\left({i}_{j,2} {Q}_{yrc}\right)- {\propto }_{kp}Q- {\propto }_{kp}*Q$$

The general utility of the ML-CPIDS framework is represented by Eq. (6): $FU$. The success of security tasks is shown by the net output against the input utility, which is captured by $N \left({U}_{out}^{j}- {U}_{in}^{j}\right)$. The term shows the impact of real-time cryptographic answers $\left({i}_{j,2} {Q}_{yrc}\right)$, and the removal of normal cryptographic overheads is shown in ${\propto }_{kp}Q- {\propto }_{kp}*Q$.

7 $$g\left(y\right)= {b}_{0}+ {b}_{1}{y}^{1}+ {b}_{2}{y}^{2}+{b}_{3}{y}^{3}+\dots + {b}_{u-1}{y}^{u-1}mod \left(q\right)$$

In Eq. (7), the cryptographic key or function is formed by the coefficients $g\left(y\right)$ and the associated terms of the polynomial, denoted as ${b}_{1}{y}^{1}$. The values are guaranteed to remain inside a finite field by modulo ${b}_{u-1}{y}^{u-1}mod \left(q\right)$, which improves security.

8 $$g\left(y\right)+g\left(0\right)= {\sum }_{k=0}^{u-1}{z}_{k}{m}_{k} \left(y\right) \times mod \left(q\right)+ {\sum }_{k=0}^{u-1}\frac{{y}_{j}}{{y}_{j}-{y}_{k}}$$

To improve security via modular operations, Eq. (8) $g\left(y\right)+g\left(0\right)$ represents the sum of cryptographic terms, whereas ${z}_{k}{m}_{k} \left(y\right)$ and $mod \left(q\right)$ represent the polynomial function and its base value, respectively. The expression describes the interaction between various variables $\frac{{y}_{j}}{{y}_{j}-{y}_{k}}$, which is essential for preserving the security and integrity of the V2X communications network in VANETs.

9 $$f\left(bQ,P\right)=f\left(Q+Q+\dots +Q,R\right)=f\left({Q}^{b}, {R}^{c}\right)+f{(Q,R)}^{bc}$$

The repetition of the cryptography function $f\left(bQ,P\right)$ is represented by Eq. 9$f\left(Q+Q+\dots +Q,R\right)$, and the additional cryptographic parameters are denoted by $f\left({Q}^{b}, {R}^{c}\right)$. The phrase $f{(Q,R)}^{bc}$ reflects the combined interaction of these operations, whereas the compounded impact is shown.

10 $$h\left(y\right)= {\sum }_{j=1}^{u}\left(h\left({y}_{j}\right)mod q\right)* {m}_{j\left(y\right)} mod q, L=h\left(0\right)mod q$$

Within the ML-CPIDS framework, Eq. (10) represents a modular mathematical method. In this case, the general cryptographic function is denoted as $h\left(y\right)$, and the modular components are denoted as $\left(h\left({y}_{j}\right)modq\right)$, with the associated terms being ${m}_{j\left(y\right)} mod q$. While maintaining the modular limitations, the summing guarantees that all the components contribute to the final cryptographic value. The baseline component value is denoted by $L=h\left(0\right)modq$.

11 $$Q{L}_{j}= {T}_{j}* {h}_{2} \in {H}_{2}+ {N}':\left\{{wje}_{j}, {U}_{t}, D\left(j,k\right), {\forall }_{k}, JW, {ql}_{j}\right\}$$

The outcome of the cryptographic operation on ${T}_{j}$ and ${h}_{2}$ is guaranteed to be ${H}_{2}+ {N}'$ in the cryptography space ${wje}_{j}$ by Eq. (11), $Q{L}_{j}$. The variables ${U}_{t}, D\left(j,k\right)$, universal quantification ${\forall }_{k}$, and $, JW, {ql}_{j}$ are included in the additional components that contribute to the cryptographic context.

12 $$f\left({h}_{2}, {\propto }_{k}\right)=f \left(qlj, I \left(D\left(j,k\right)\right)\right)+f \left({h}_{2}, {\partial }_{\propto }\right)=f\left({qj}_{p}, I \left(D\left(j,k\right)\right)\right)$$

The cryptographic function that guarantees resilience in cryptographic operations is represented by Eq. (12), $f\left({h}_{2}, {\propto }_{k}\right)$, which involves $f$ and the security parameter ${\propto }_{k}$. The cryptographic operations using $qlj, I \left(D\left(j,k\right)\right)$ are denoted by $f \left({h}_{2}, {\partial }_{\propto }\right)$, whereas the cryptographic validations of integrity involving $D\left(j,k\right)$ are represented by ${qj}_{p}$.

13 $$f\left({h}_{2}, {\forall }_{k}\right)=f\left({h}_{2}, I\left(D\left(j,k\right)\right)*{T}_{j}\right)+f \left({QL}_{j}, I \left(D\left(j,k\right)\right)\right)$$

To ensure that cryptographic operations work for all important variables $f\left({h}_{2}, {\forall }_{k}\right)$, Eq. (13) represents the cryptographic function that uses ${h}_{2}$ and universal quantification $D\left(j,k\right)$. To improve the security and dependability of the cryptographic process, the combined digital integrity check with ${T}_{j}$ is denoted by the expression ${h}_{2}, I\left(D\left(j,k\right)\right)$. By combining the encryption operation, ${QL}_{j}, I \left(D\left(j,k\right)\right)$ ensures that cryptographic keys and data are handled securely in V2X communication for VANETs.

14 $$f\left({h}_{2}, {\forall }_{Aggr}\right)=f\left({h}_{2}, I \left({Su}_{j}\right)\right)+\left({T}_{1}+ {T}_{2}+ {T}_{3}+\dots + {T}_{u}\right)$$

The encryption function involving ${h}_{2}$ and aggregation over all entities indicated by ${\forall }_{Aggr}$ is represented by Eq. (14), $f\left({h}_{2}, {\forall }_{Aggr}\right)$. The cryptographic integrity check guarantees the security and correctness of the aggregated data, and the phrase $f\left({h}_{2}, I \left({Su}_{j}\right)\right)$ reflects this. Furthermore, the combined cryptographic techniques ${T}_{1}$, which contribute to overall improvement in security, are represented as ${T}_{1}+ {T}_{2}+ {T}_{3}+\dots + {T}_{u}$.

15 $$f\left({ql}_{Aggr}, I \left(S{u}_{j}\right)\right)=f \left({T}_{1}+ {\sum }_{k=1}^{u}{\forall }_{m+k} \left(r+st\right)-\left(v-mt\right)\right)$$

The cryptographic function that guarantees the integrity of the aggregated data ($S{u}_{j}$) is represented by Eq. (15), ${ql}_{Aggr}$. This function ensures that the aggregated ${T}_{1}$ values (${\forall }_{m+k}$) and aggregated $r+st$ are equal. Together, the cryptographic operations $\left(r+st\right)-\left(v-mt\right)$ represent an effort to strengthen security.

16 $$RS=\left(x1*mUN\right)+\left(x2*pTn\right)+\left(x3*pGN\right)+\left(x4*pDT\right)$$

The analysis of privacy, denoted as $RS$, is calculated by multiplying individual metrics: in Eq. (16), $mUN$ for user mobility, $pTn$ for traffic noise, $pGN$ for geographical location, and $pDT$ for data transmission, with weighted factors $x1$, $x2$, $x3$ and $x4$ as inputs.

17 $$Jp{e}_{1}+Jp{e}_{2} =i\left(e\right)mod P+\left({Jpe}_{1}\approx i \left(Fingerprint\left(e\right)\right)mod P\right)$$

Equation (17) denotes the analysis of security through $Jp{e}_{1}$ and $Jp{e}_{2}$, which represent parts that contribute to channels of secure communication. To ensure data correctness and protection against tampering, cryptographic integrity tests are represented by $i\left(e\right)mod P$. As a security mechanism, fingerprinting is introduced by the phrase $\left({Jpe}_{1}\approx i \left(Fingerprint\left(e\right)\right)mod P\right)$ to uniquely identify individuals or datasets, improving authentication and preventing unwanted access.

18 $$\left(\left(p\right){U}_{tn-f}+\left(m\right){U}_{i}\right)+\left(\left(p+2\right){U}_{tn-f}\right)+\left(2p\right){U}_{ps-f-q}+\left(p+1\right)$$

The analysis of threat detection is represented by Eq. (18), where $U$, such as $\left(p\right){U}_{tn-f}$ for anomalies in the traffic network, $\left(m\right){U}_{i}$ for the detection of intrusions and $\left(p+2\right){U}_{tn-f}$ for packet security. The weighted factors $\left(2p\right){U}_{ps-f-q}$ and $\left(p+1\right)$ combine these components, highlighting their combined function in detecting and reducing different threats.

19 $$qj{d}_{j}=f\left({\sum }_{j=1}^{w}{\forall }_{j}+ {i}_{2}(qj{e}_{j}^{1}|\left|qj{e}_{j}^{2}\right||tup){i}_{3}({n}_{j}||vt, {Q}_{UB})\right)$$

The analysis of traffic safety is denoted as $qj{d}_{j}$ and is a product of several factors, including ${\forall }_{j}$ for universal quantification, ${i}_{2}(qj{e}_{j}^{1}|\left|qj{e}_{j}^{2}\right||tup$ for secure protocols and vehicle-to-vehicle communication and ${i}_{3}({n}_{j}||vt, {Q}_{UB})$ for the detection of attacks and vehicle surveillance in Eq. (19).

20 $$f\left(bQ, CP\right)={f (Q,R)}^{bc}+f \left(Q,Q\right)\ne 1-f \left({\forall }_{j}, Q\right)=f\left(I\left(UJ{E}_{j}\right)\right)$$

Equation (20), $f\left(bQ, CP\right)$, represents the analysis of efficiency in cryptographic operations ($bQ$) with thorough policy enforcement ($CP$). The statement ${f (Q,R)}^{bc}$ shows that cryptographic balances and checks are used iteratively to strengthen security measures. The fact that $f \left(Q,Q\right)$ ensures resilience in data verification and access control $f\left(I\left(UJ{E}_{j}\right)\right)$ speaks to the intricate relationship between cryptographic effectiveness and universal quantification.

Algorithm 2 describes a complete assessment of an idea proposed for intrusion detection through a designed methodology. It begins with data collection: the backbone that holds the whole process together, followed by features and their respective labels, extracted from the data collection process, for model training purposes.

The algorithm preprocessing the training data splits it into separate training and testing datasets. Now, the model fits the training data. To assess communication efficiency in this direction, it calculates the cost from the process. Then, this model is tested on the test set, to obtain the predictions. It measures performance based on accuracy, precision, recall, and F1 score, which identifies the algorithm’s robustness in detecting threats and ensuring data security. It established a structured approach not only to assess the performance of the models but also to present areas for improvement in intrusion detection systems.

**Algorithm 2:**
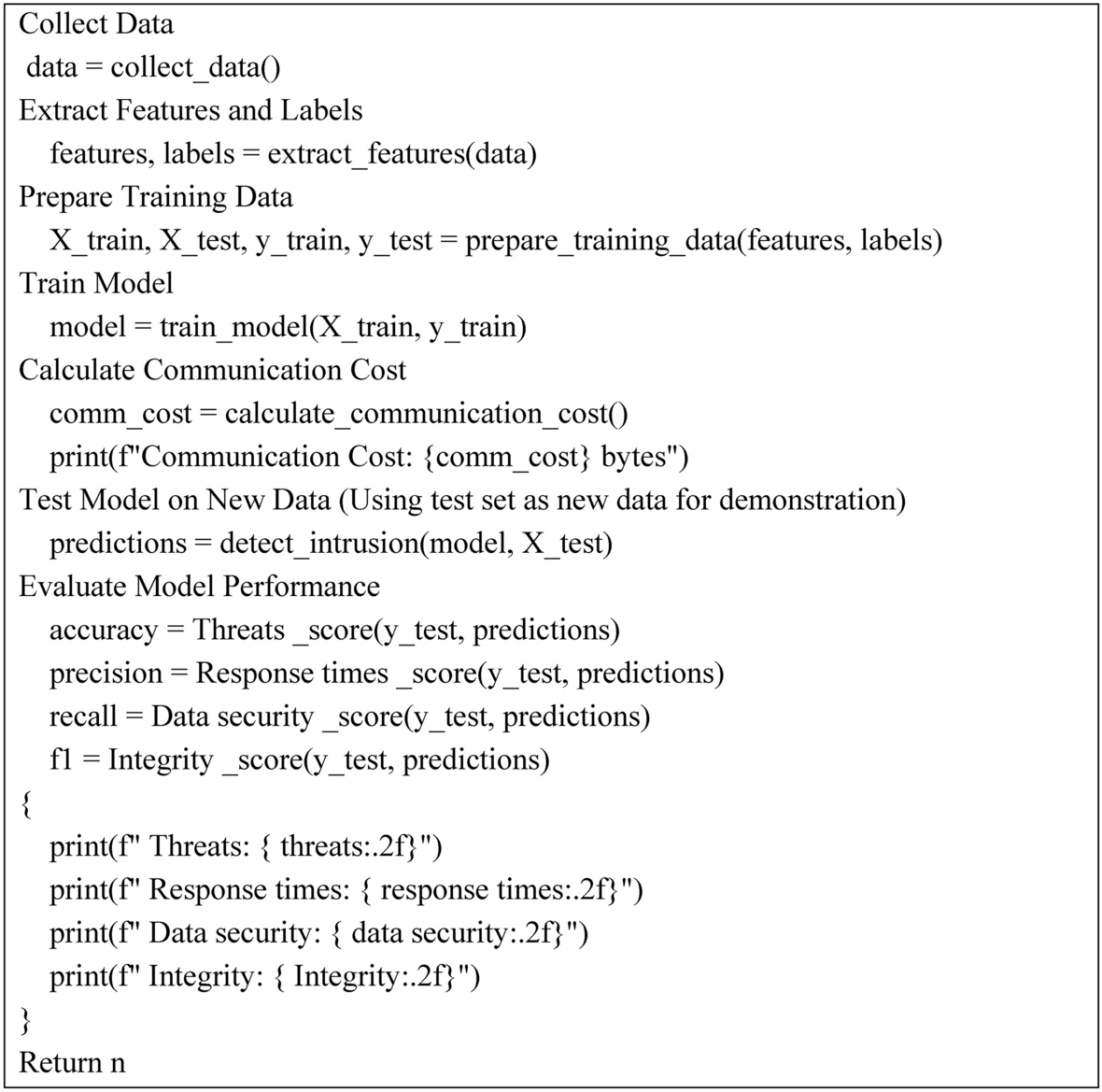
For evaluation of the proposed method.

The effectiveness of the ML-CPIDS in various VANET settings is shown through extensive simulations, emphasizing improvements in safety, confidentiality, and threat detection. By presenting a robust method to address commonplace security and privacy troubles, the suggested era has accomplished effects for networks of self-sustaining cars, traffic management in metropolitan regions, and different vehicular communication structures.

## Results and discussions

The development and deployment of intelligent transportation structures relies heavily on VANETs, which enable V2X connectivity. Ensuring privacy, integrity, effectiveness, and protection of these interactions is paramount. The proposed ML-CPIDS, or machine learning-cryptographic protocol-based integrated detection system, offers a novel technique for VANET development via the integration of machine learning cryptographic protocols.

### Dataset description

Connectivity powers the transition toward automated mobility. Future Cooperative Intelligent Transportation Systems (C-ITS) will connect vehicles, signals, infrastructure, and vulnerable users for improved safety and efficiency. WLAN-based peer-to-peer (IEEE 802.11p, ITS-G5 in Europe) and cellular-based C-V2X compete for V2X connectivity^30^. Regardless of communication standards, common message interfaces are essential for vehicle communication, particularly across manufacturers. V2AIX is a new multimodal real-world dataset of ETSI ITS communications from public road traffic. Over 230,000 V2X communications from 1800 vehicles and roadside units in public road traffic have been collected via measurement drives and fixed infrastructure. This allows analysts to evaluate real-world V2X data and use standardized V2X messages in ROS-based autonomous driving applications.

The computing facility used in ML-CPIDS research is a combination of high-performance computing servers and edge computing devices built to analyze data in real time for vehicle networks. The technical specifications for fast data transfer can differ but often include multicore CPUs, enough random-access memory, and reliable network connections. Here, the Python tool was used for analysis. The ML-CPIDS framework in a VANET environment has demanding standards, and these languages provide a compromise between high performance and ease of development.

**Fig. 5:**
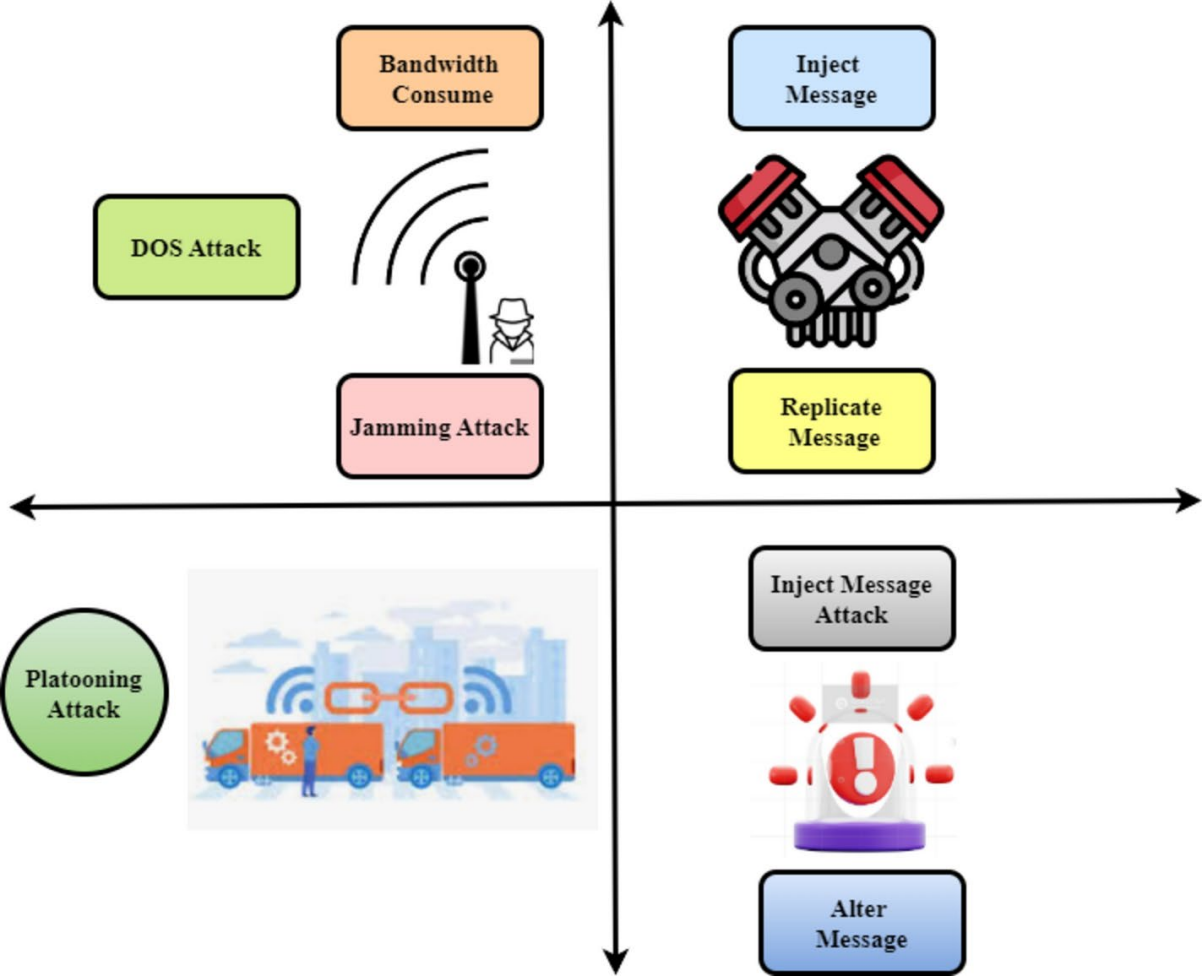
Attacks on the V2X Network.

Figure 5 illustrates the attacker model that focuses on disrupting traveler comfort and safety, highlighting the potential attacks within the V2X network. An antenna’s illusion attack leads the sensor to capture road condition data for warning signals, creating an illusion for nearby cars^22^. As a master node in the intervehicle communication network, a malicious vehicle may conduct an inject message attack by producing many copies of the same message or constructing a new message by injecting harmful information and changing the originals. The image depicts many vehicle network assaults, particularly on V2X communication systems. DoS and jamming may overwhelm or close communication channels, preventing infrastructure‒vehicle data exchange. Adding or repeating deceptive information might cause cars to make risky decisions based on inaccurate data. Platooning attacks occur when many vehicles move in sync, and a disruption might cause an accident. Each assault type is risky, emphasizing the need for complete safety safeguards in connected automobile systems.

By using an adaptive machine learning model, the ML-CPIDS system can identify threats across heterogeneous networks, regardless of the data type or context. To ensure a consistent level of security and continuous communication between infrastructure and vehicles, the ML-CPIDS follows the standards and uses flexible design concepts. Furthermore, it combines machine learning with a modern cryptographic algorithm and provides robust authentication that can adjust to threats and network circumstances.

In ML-CPIDS, end-to-end encryption prevents eavesdropping by encrypting data at the source and allowing only the intended receiver to decipher it. Since it uses differential privacy and homomorphic encryption, the ML-CPIDS may analyze data without disclosing sensitive information. Security assessments and upgrades help react to changing threats and maintain confidentiality. Owing to these procedures, the VANET system has a thorough strategy to secure critical data.

Users and regulatory bodies can depend on machine learning-powered threat detection options. The system compares characteristic significance to identify which information affects the detection results. Openness and engagement enable stakeholders to influence system development via user feedback and model performance upgrades. Audits and compliance increase the security credibility of the ML-CPIDS.

## Simulation-based security analysis for threat detection

The following section provides a comprehensive analysis of the proposed ML-CPIDS method, focusing on privacy, authentication, latency, threat detection, efficiency, and integrity of the system.

### Analysis of privacy

Protecting private information, including position data, identities, and communication patterns, in vehicle networks is an important function of privacy analysis in systems such as ML-CPIDS. Without adequate security measures, vehicles that use V2X communication expose themselves to privacy breaches due to the massive volumes of data exchanged between vehicles and roadside infrastructure. Ensuring that no unauthorized parties can employ these data is the objective of privacy analysis.

**Fig. 6:**
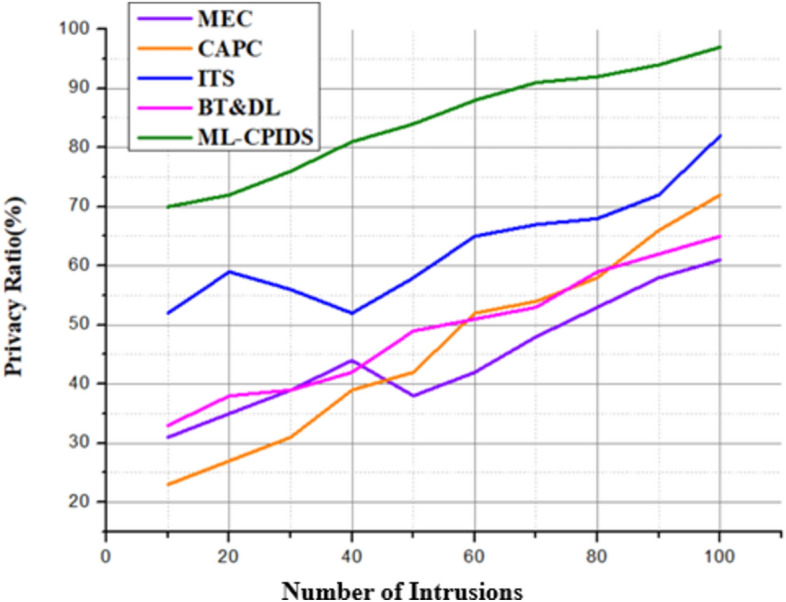
Privacy analysis.

The ML-CPIDS method greatly increases the privacy of VANETs in V2X communication, as shown in Fig. 6 and Eq. (16). A combination of advanced cryptographic protocols and machine learning software ensures robust authentication, ensures data transmission and can identify threats in real time. When used together, these safeguards restrict unknown individuals from seeing, changing, or listening to drivers’ confidential data. The capacity of the ML-CPIDS system to spot risks in real time and its ability to react dynamically to new threats contribute to an improvement in privacy by preventing unauthorized access and preserving data integrity.

ML-CPIDS for VANETs reportedly achieves 98.2% privacy, which is most likely the combination of results such as differential privacy, anonymity set size, and privacy leakage. An adversary’s ability to determine sensitive information (such as a vehicle’s identity or position) from the system is a metric of privacy leakage; entropy-based analysis is a common tool for quantifying this risk. The anonymity set size measures the effectiveness of user-generated masking of individual automobiles in a crowd, making it difficult for attackers to identify individual vehicles. To ensure that the system cannot access any user-specific information, differential privacy techniques such as introducing noise into the data or outcomes are used. Even in the unpredictable and latency-sensitive environment of V2X communication on VANETs, these measures have been refined to achieve 98.2% privacy, protecting user data without significantly lowering the communication speed.

### Analysis of authentication

**Fig. 7:**
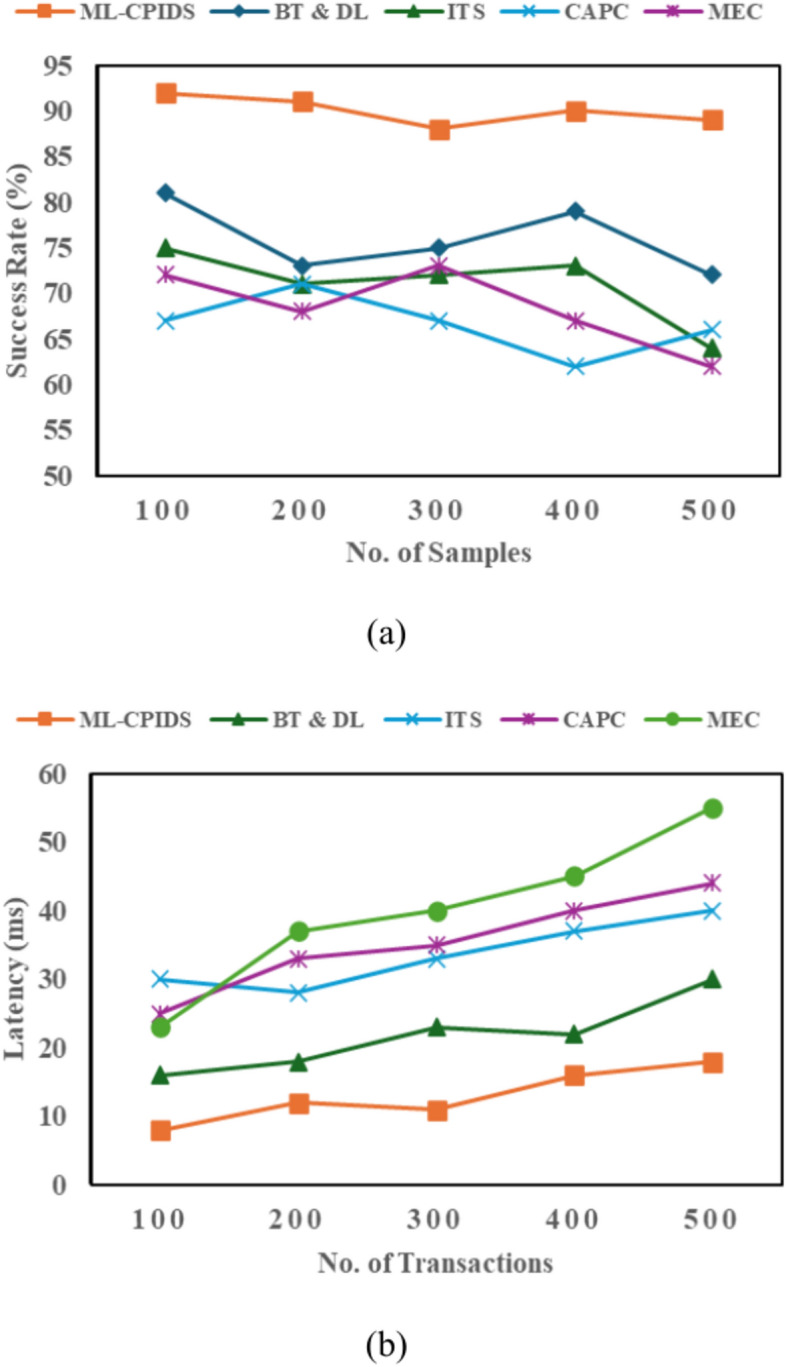
Authentication Analysis. (**a**) Authentication success rate, (**b**) latency.

Figure 7 (a) and (b) represent the authentication success rate and latency, respectively, based on Eq. (17) recommended for VANET communication authentication. This involves the utilization of authentication with the ML-CPIDS through the recommended procedure. The classical VANET authentication solutions use cryptographic methods such as digital certificates or signatures to verify the identities of vehicles and community entities. In addition, it couples modern cryptographic protocols with machine learning to provide strong authentication that can adapt to network conditions and threats.

Additionally, machine learning can analyze communication patterns for spoofing detection and illegal access attempts. As a result, the ML-CPIDS enhances authentication through real-time response and supervision. This approach authenticates communications/users while adjusting appropriate levels of authentication in the face of security threats. Because of the cryptographic algorithms, which are designed for processing authentication requests quickly without sacrificing security, the ML-CPIDS performs with minimal latency. ML algorithms in ML-CPIDS can quickly identify patterns and make predictions to speed up the authentication process, lowering the computing load and minimizing delay. This method guarantees quick and precise authentication, enabling the system to process more transactions more effectively.

### Analysis of threat detection

**Fig. 8:**
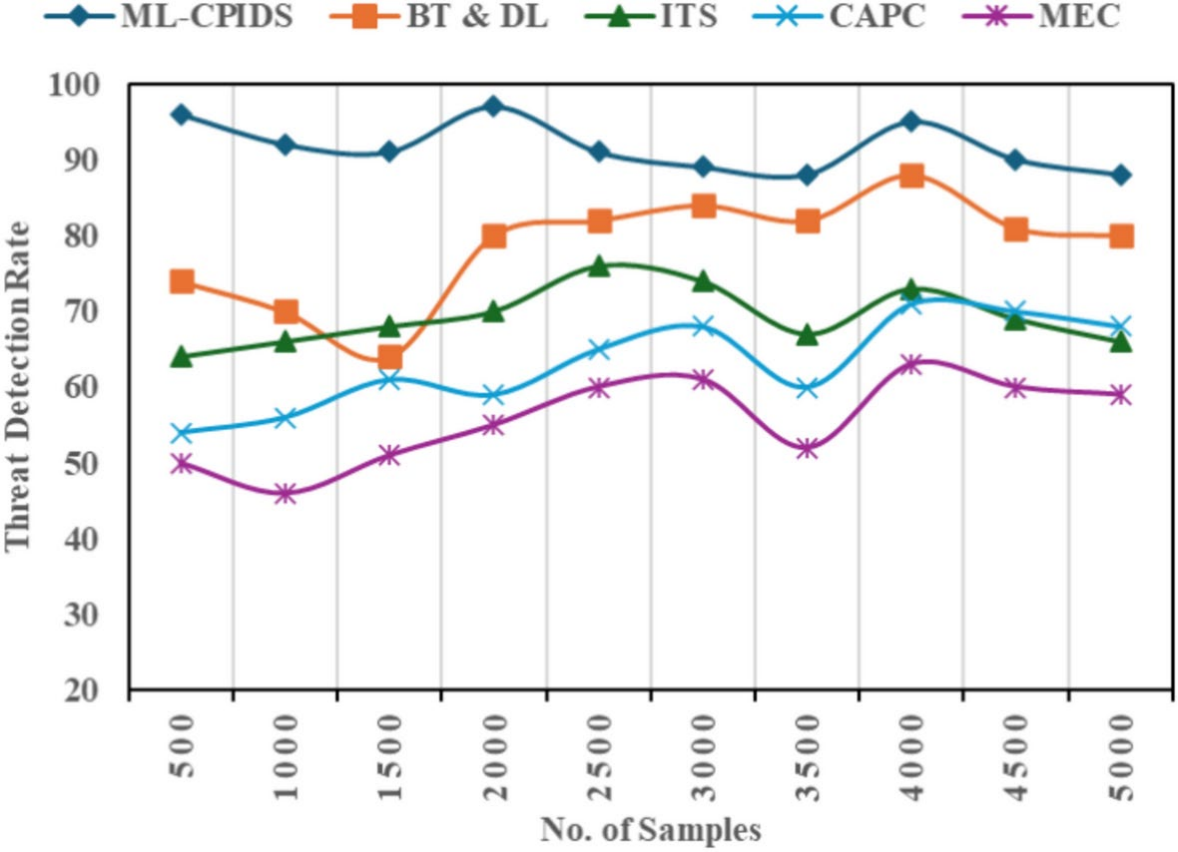
Threat detection rate.

ML-CPIDS uses machine learning techniques to identify threats in VANETs in real time, as shown in Fig. 8 and Eq. (18). It continuously watches V2X traffic to detect attacks, abnormalities, and potential security breaches quickly. ML-CPIDS guarantees reliable and secure communication between V2X devices by detecting and avoiding issues before they escalate. Reliable threat detection in VANET systems is achieved by its capacity to adapt to new attack vectors and its efficiency in recognizing known and novel threats. With this proposed method, threat detection analysis yields an average value of 92%.

### Analysis of efficiency

**Fig. 9:**
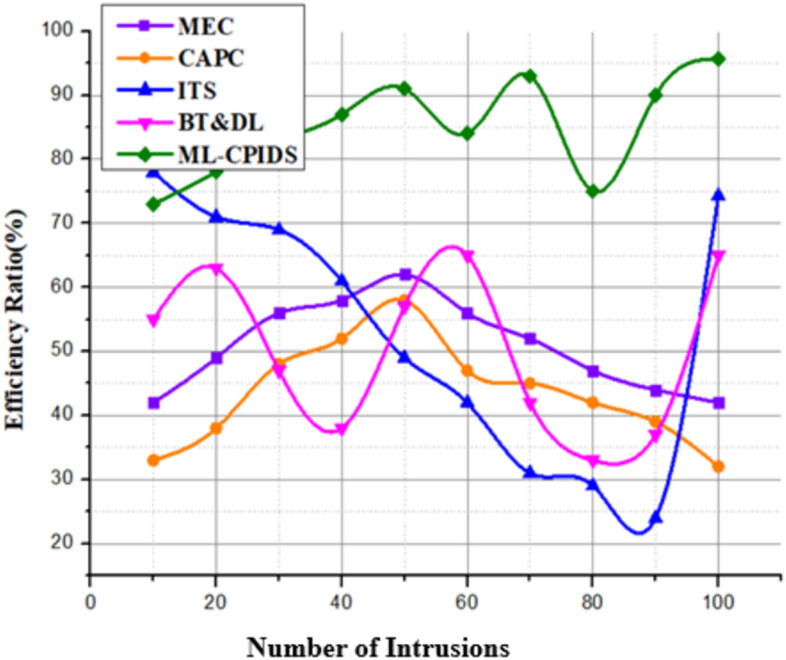
No. of Intrusions vs. Efficiency.

ML-CPIDS combines machine learning with cryptographic protocols to display its efficacy, as demonstrated in Fig. 9 and Eq. (19). The efficiency of the V2X communique in VANETs has progressed via this integration. The approach simultaneously reduces the important computational cost while maintaining strong safety mechanisms such as authentication and encryption. Due to its adaptive ability and real-time data analysis ability, the ML-CPIDS enhances the general performance of the V2X communique and is therefore suitable for use in dynamic visitor conditions. In addition, the system’s simulation results show that it can feature well in special VANET scenarios. This guarantees secure and dependable conversation between vehicles and roadside infrastructure. This proposed approach analyzes performance and obtains 95.7% accuracy.

### A systematic analysis of message and data integrity

**Fig. 10:**
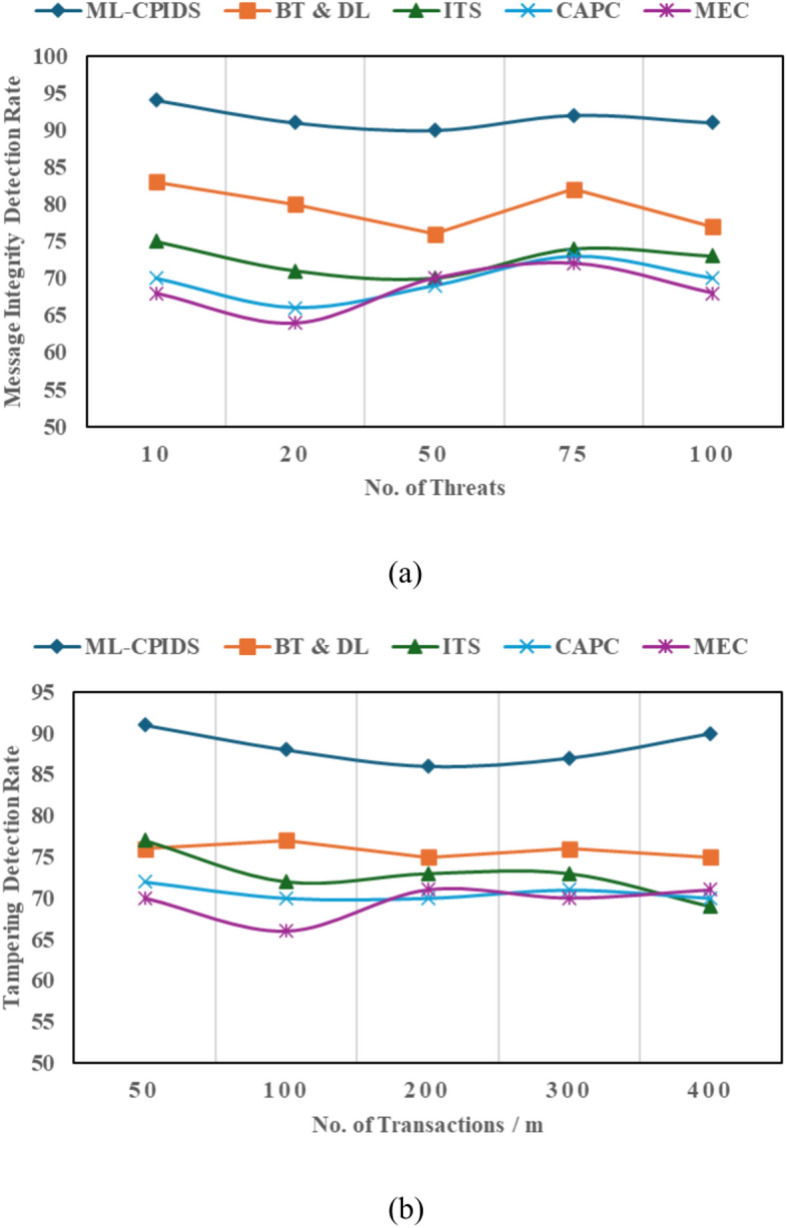
Integrity analysis (**a**) message integrity analysis (**b**) tampering detection analysis.

The process of integrity analysis for verifying VANET messages and data is explained through Eq. (20) and Figs. 10(a) and (b). The Message Integrity Detection (MID) and Tampering detection (TD) rates are measures that are related to data integrity. However, they focus on different aspects of integrity in the network. The MID detects the changes in the messages themselves, whereas TD detects tampering at a transactional or packet level. The MID focuses on content-specific parameters such as hash values, signatures, and message payloads. TD focuses on a transactional parameter such as timestamps, packet headers, or network protocol metadata.

VANETs, where vehicles and infrastructure communicate to improve traffic efficiency and road safety, require data integrity to avoid tampering and send trustworthy information. VANET integrity checks use hash functions and digital signatures. Data integrity is maintained during transmission via these techniques. Injection attacks and data manipulation threaten VANETs. Modern VANET security frameworks use real-time monitoring and complex algorithms for integrity analysis. These systems continually scan data packets for manipulation. Machine learning may be used to recognize data patterns that suggest integrity breaches and take proactive steps to reduce risks and preserve the VANET connection.

This proposed approach significantly enhances privacy and security by providing robust authentication, secure data transmission, and real-time chance identification. To offer a straightforward and efficient communication framework for VANETs, ML-CPIDS plans to cope with these troubles. This will help create technologically more secure and safer traffic situations in the long run.

ML-CPIDS can recognize and stop real-time threats better than cryptographic methods or isolated machine learning. ML-CPIDS analyzes large amounts of data in real time to find patterns and outliers that can indicate an attack, unlike static rules or signatures, which can be used to identify new threats. The connection enables ML-CPIDS to launch data-driven cryptographic measures and provide more dynamic security responses. The safety measures of the ML-CPIDS are continually adjusted to recognize threats, decrease response times and ensure data security and integrity. This automates the encryption algorithm and key improvements. Compared with conventional systems, machine learning and cryptographic protocols provide ML-CPIDS with a proactive and adaptive security architecture that responds faster to real-time attacks.

## Conclusion

The ML-CPIDS effectively combines advanced cryptographic protocols with machine learning to enhance security in V2X communication. It offers strong authentication, encryption, and real-time threat detection capabilities, ensuring sensitive information remains protected. By integrating robust cryptographic techniques, this approach addresses key privacy and security challenges in VANET networks. The proposed system demonstrates a practical solution for improving the safety and reliability of V2X communication. Simulations in various VANET scenarios have proven this solution effective, leading to significant improvements in privacy, security, and threat detection. The ML-CPIDS approach outperforms existing methods by providing stronger privacy, authentication, lower latency, and enhanced threat detection for V2X communication in VANET networks. This paper also examines the scalability of the ML-CPIDS to support the increasing number of connected vehicles. To make ML-CPIDS even better, analysts might specialize in various key regions in the future. The initial step is working into quantum cryptography, which might offer more steady processes. ML-CPIDS could be tested in real-time situations via preliminary tasks and collaboration with vehicle manufacturers and city planners. This will eventually result in its wider utility in smart transportation structures. Even with its strong multilayer security architecture, ML-CPIDS could be susceptible to data poisoning and other potential vulnerabilities that adversaries can exploit. To improve the overall security of the ML-CPIDS, the system should have rigorous data validation procedures to prevent data poisoning and advanced behavioral analytics to improve insider threat detection.

## Data Availability

The data that support the findings of this study are openly available in https://datasetsearch.research.google.com/search?src=0&query=V2X%20Communication%20&docid=L2cvMTF2d3k4Y19neQ%3D%3D.
